# Anatomical Analysis of Mandibular Posterior Teeth using CBCT: An Endo-Surgical Perspective

**DOI:** 10.14744/eej.2021.40427

**Published:** 2021-12-21

**Authors:** Shehabeldin SABER, Shaimaa Abu EL SADAT, Alya TAHA, Nawar Naguib NAWAR, Adham Abdel AZIM

**Affiliations:** 1. Department of Endodontics, The British University Faculty of Dentistry, Egypt; Department of Endodontics, Ain Shams University, Cairo, Egypt; 2. Department of Oral and Maxillofacial Radiology, Ain Shams University, Cairo, Egypt; 3. Department of Endodontics, Ain Shams University, Cairo, Egypt; 4. Department of Endodontics, The British University Faculty of Dentistry, Egypt; 5. Department of Endodontic, University of the Pacific, CA, USA; aazim@pacific.edu

**Keywords:** Endodontic surgery, mandible, mandibular canal, mental foramen, microsurgery, osteotomy, posterior teeth, root resection

## Abstract

**Objective:**

This study sought to analyse the relationship between mandibular posterior teeth and the surrounding anatomical structures.

**Methods:**

A total of 170 CBCT images were examined to obtain measurements regarding the following: buccolingual (BL) and mesiodistal (MD) root thickness at the standard level of resection (3 mm from the apex), the thickness of the overlying buccal and lingual bone at the same level, the proximity of the mandibular canal (MC) to the apices of the mandibular posterior teeth, as well as the horizontal location of the mental foramen (MF).

**Results:**

The BL root width at 3 mm from the apex was the broadest at the mesial roots of the first molars with males: 5.33±0.99 mm and females: 5.16±0.88 mm (mean±SD). The root width was narrowest at the second premolars (males: 3.80±0.83 mm; females: 3.61±0.60 mm). At the same level; the buccal bone was thickest over the distal roots of the second molars (males: 6.92±1.85 mm; females: 6.95±1.95 mm) and thinnest over the first premolars (males: 1.73±0.93 mm; females: 1.49±1.01 mm), while the lingual bone was thickest over the distal roots of the first molars (males: 5.58±1.36 mm; females: 4.52±1.24 mm) and thinnest over the distal roots of the second molars (males: 3.13±1.50 mm; females: 2.60±1.46 mm). The nearest root apices to the MC were the distal roots of the second molars (male: 1.21±1.45 mm; female: 1.75±1.97 mm), while the furthest were the mesial roots of the first molars (male: 4.00±2.39 mm; female: 4.77±2.58 mm). The most common horizontal location of the MF was between the first and second premolars (51.8%). The lingual bone was significantly thinner over both roots of first molars in females (P<0.05).

**Conclusion:**

As the position of the teeth became more posterior, the buccal bone thickness increased, the lingual bone thickness decreased, and the distance to the MC became closer. CBCT analysis provides distortion- and superimposition-free images of the relevant anatomic structures.

## Introduction

When nonsurgical root canal treatment or orthograde retreatment is not appropriate or fails, endodontic microsurgery (EMS) becomes the treatment modality of choice to address persistent apical periodontitis (1, 2). EMS has been shown to have a high success rate and a predictable outcome (3). However, EMS in the mandibular posterior region can be technically challenging, possibly due to the limited visibility and accessibility, the considerable thickness of the buccal cortical plate, the lingual inclination of the roots, and their proximity to the mandibular canal (MC) (4). In addition, Libersa et al. (5) illustrated a higher incidence of procedural errors and a higher probability of persistent neurosensory disturbances with root-end surgeries in the posterior mandible. Thus, some clinicians deter from EMS in the posterior mandible to avoid these complications, despite recent improvements in the surgical armamentaria (6).

**HIGHLIGHTS**

• CBCT is a dependable, precise tool for preoperative anatomic analysis of the surgical site during planning Endodontic Microsurgery.
• As the position of the teeth become more posterior, the buccal bone thickness increases, while both the lingual bone thickness and the distance to the mandibular canal decrease.
• As the position of the planned apical surgery goes posteriorly, alternative options, such as intentional replantation and bony lid technique, should be considered.

Cone-beam computed tomography (CBCT) is a valuable tool in EMS pre-surgical assessment and treatment planning (4). It allows for three-dimensional reconstruction of the dento-maxillofacial complex in an accurate 1:1 anatomic representation (7) while exposing the patient to a low radiation dose (8), thus acquainting the surgeon with the anatomic landmarks and structures adjacent to the surgical site as well as tooth dimensions and anatomy. Therefore, CBCT has been recommended as the imaging modality of choice for pre-surgical assessment by the American Association of Endodontists, the American Association of Oral and Maxillofacial Radiology and the European Society of Endodontics (ESE) (8, 9). However, CBCT is not always available or affordable for the patients (10). Therefore, descriptive morphologic studies are required to provide information about the relationship between mandibular posterior teeth and the surrounding anatomical structures (4, 11). Such data is notably lacking for the Egyptian population. Therefore, we aim in this study to use CBCT to:

1. Acquire normative information regarding the buccolingual (BL) root thickness and the thickness of the overlying buccal and lingual bone at the 3 mm resection level,
2. The proximity of the MC to the apices of the mandibular posterior teeth,
3. The horizontal location of the MF, and,
4. Compare the measurements between male and female patients.

The null hypothesis was there was no difference between the tested groups.

## Materials and Methods

### Sample size calculation

A power analysis was performed to apply a two-sided statistical test with an alpha level of .05 and beta levels .95, and an effect size of 1.24, calculated based on the results of Jeon et al. (12), the predicted sample size (n) was a total of (36) cases. The sample size was increased to 170 cases. The sample size calculation was performed using G*Power version 3.1.9.7.® (Heinrich-Heine-Universität, Düsseldorf, Germany).

### Subjects

A research Ethics Committee approved the study in Ain Shams University (Protocol number: FDASU-RecEM061705). CBCT images of 170 patients were included in the study. Scans were collected from a private maxillofacial imaging centre along with the demographic data of the anonymous patients and were acquired using a CBCT machine (Cranex PP3-1; Soredex, Tuusula, Finland) with exposure settings of 90 kV, 10 mA, 6.1 seconds, a field of view of 6×8 cm (one side of the mandible), and voxel size: 200 μm.

### Inclusion and exclusion criteria

All included patients had to have all their mandibular posterior teeth on the examined side, except for the third molars, i.e., four posterior teeth were examined per patient. Patients with radiographic evidence of periapical lesions, periodontal disease, resorbed roots, immature molars, or mixed dentition were excluded. A periapical lesion was defined as a periapical radiolucent area that was in contact with the radiographic apex of the root and measured at least twice the width of the periodontal ligament space (13). Periodontal disease was identified according to its earliest signs as a break in the continuity of lamina dura and a wedge-shaped radiolucent area at the mesial or distal aspect of the periodontal ligament space (14). A resorbed root was detected when three authors, including the radiologist, had a consensus (15).

### Calibration and measurements

All data from the CBCT examinations were acquired in a digital DICOM format, imported to OnDemand3D® App software (Cybermed, Seoul, Korea), and viewed on an 18.5-inch HD LED monitor with a resolution of 1366×768. Three examiners (two endodontists and one oral radiologist with more than 10 years of experience) evaluated all the scans twice. The examiners were calibrated at the beginning of the study by evaluating 15% of the scans, and the interclass correlation coefficient (ICC) scores were determined (ranged from 0.87-0.92, with a 95% confidence interval). A break was taken after evaluating 3 consecutive scans to avoid eye strain. The examiners could change the viewer settings such as contrast, density, and sharpness. In addition, they were able to magnify the images for better identification and visualisation of the measured structures.

**Figure 1.**
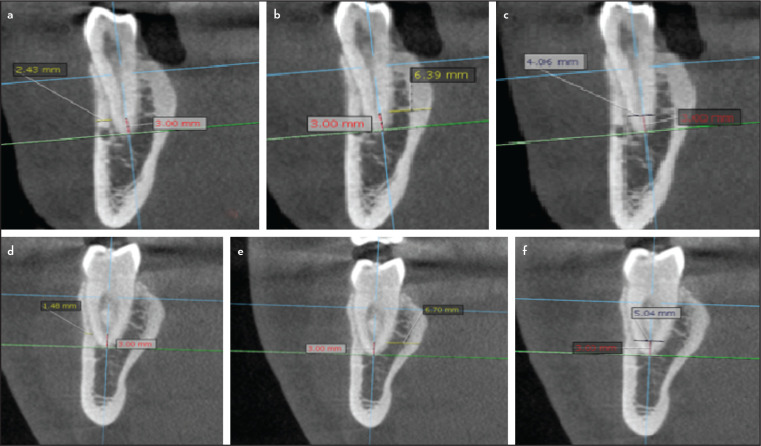
Anatomical measurements done after Reference planes (axial, coronal and sagittal) calibration for a premolar (a-c) and a molar (d-f) as detailed in the methodology

**Figure 2.**
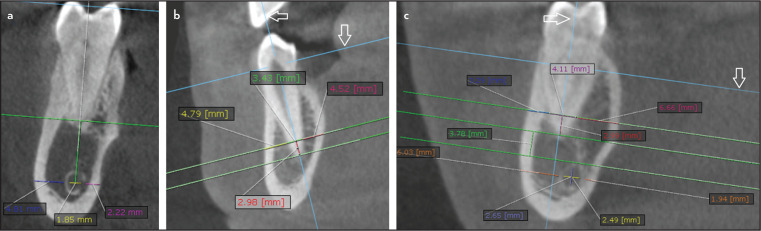
Representative samples of the extensive images’ analysis done: (a) Mandibular canal diameter: 1.85 mm, buccal and lingual bone thicknesses relative to the mandibular canal: 4.81 and 2.22 mm, (b) Arrows denote reference planes. Root thickness at resection level: 3.43 mm, buccal and lingual bone thicknesses relative to the root at resection level: 4.79 and 4.52 mm respectively, (c) Arrows denote reference planes. Root thickness at resection level: 4.11 mm. Buccal and lingual bone thicknesses relative to the root at resection level: 3.29 and 6.66 mm respectively. Horizontal and vertical diameters of the mandibular canal: 2.65 and 2.49. Buccal and lingual bone thicknesses relative to the mandibular canal: 6.03 and 1.94 mm respectively. Distance between the apex and mandibular canal: 3.78 mm

Measuring the buccal and lingual bone thickness and root dimensions 3 mm from the apex (Figs. 1, 2)

For the premolars, the coronal plane was realigned to divide the tooth into mesial and distal halves and the sagittal cut was adjusted to be passing through the buccal cusp tip and the root apex. For the molars, the coronal cut was again adjusted to divide the tooth mesiodistally and the sagittal cut was adjusted to be passing through the central fossa and the root apex.

For premolars, the axial plane was first adjusted to pass through the cementoenamel junction on the axial view. Next, the reference planes were adjusted so that the sagittal plane bisects the tooth BL and the coronal plane bisects the tooth MD. Next, the axial plane was adjusted for the molars below the furcation area, and each root was measured separately. Reference planes were adjusted so that the sagittal plane bisects the root BL and the coronal plane bisects the root MD.

On the sagittal view: The coronal plane was adjusted to pass through the apical third of the tooth and bisect the root M-D.

On the coronal view: For the first premolar, the sagittal plane was adjusted to pass along their long axis, passing by the root apex and the buccal cusp tip. For the second premolar, the sagittal plane was adjusted to be passing along the long axis of the tooth passing by the root apex and the central fossa, while for molars, it was adjusted to bisect the root along the long axis passing by the root apex.

### Detection of the horizontal position of mental foramen (MF)

**Figure 3.**
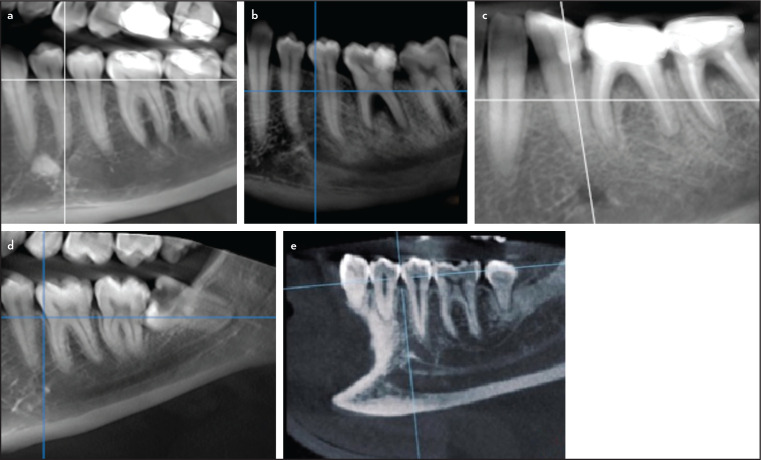
Different possibilities of mental foramen location & reference planes adjustment: (a) Directly below the mandibular first premolar, (b) Between mandibular premolars, (c) Directly below the mandibular second premolar, (d) Between the mandibular second premolar and the first molar, (e) Proper reference planes adjustments before identifying the location

The MF was detected on the reconstructed panoramic view (Fig. 3) according to the classification of Chkoura et al. (16) as follows:

1. Located between the long axis of the mandibular canine and mandibular first premolar
2. In the same line with the long axis of the lower first premolar
3. Between the long axis of the mandibular first premolar and mandibular second premolar
4. In line with the long axis of the mandibular second premolar
5. Between the long axis of the second mandibular premolar and the first mandibular molar
6. In the same line with the long axis of the mesial root of the mandibular first molar.

### Statistical analysis

Categorical data were presented as frequencies and percentages and were analysed using Fisher’s exact test. Numerical data were explored for normality by checking the data distribution, calculating the mean and median values using Kolmogorov-Smirnov and Shapiro-Wilk tests. Data showed parametric distribution and were presented as mean and standard deviation (SD) values. One-way ANOVA followed by Tukey’s post hoc test was used for the statistical analysis. The significance level was set at P≤0.05 within all tests. Statistical analysis was performed with R statistical analysis software version 4.0.3 for Windows.

## Results

A total of 170 CBCT scans (680 teeth) were evaluated. In addition, inter- and intra-reliability tests were performed and showed a high ICC score (>0.9) between the different readings. From the 170 CBCT scans, 69 (40.6%) of the patients were males, and 101 (59.4%) were females.

The total resection depth (BL root thickness and buccal cortical plate thickness), the lingual cortical plate thickness at the standard level of resection at 3 mm from the apex, and the distance between the teeth apices and the MC are presented in Table 1. The B-L root width at 3 mm from the apex was broadest at the mesial roots of the first molars (males: 5.33±0.99 mm and females: 5.16±0.88 mm (mean±standard deviation), and narrowest at the second premolars (males: 3.80±0.83 mm and females: 3.61±0.60 mm). The buccal bone was thickest over the distal roots of the second molars (males: 6.92±1.85 mm and females: 6.95±1.95 mm) and thinnest over the first premolars (males: 1.73±0.93 mm and females: 1.49±1.01 mm).

**Table 1.** Thickness of the buccal bone overlaying the mandibular posterior teeth, their buccolingual root thickness and the total resection depth needed for endodontic microsurgery

Tooth	Buccal thickness (mean±SD, mm)		Root thickness (mean±SD, mm)		Total thickness (mean±SD, mm)	
Male	Female	P value	Male	Female	P value	Male	Female	P-value
First premolar	1.49±1.01	1.73±0.93	0.119	3.76±0.72	3.90±0.71	0.216	5.26±1.24	5.63±1.11	0.042*
Second premolar	1.93±1.08	1.85±0.94	0.634	3.61±0.60	3.80±0.83	0.113	5.54±1.19	5.65±1.14	0.543
Mesial root canal of first molar	1.73±1.05	1.52±0.93	0.202	5.16±0.88	5.33±0.99	0.291	6.89±1.16	6.85±1.10	0.815
Distal root of first molar	3.03±1.36	2.41±1.31	0.006*	4.28±0.74	4.44±0.82	0.240	7.31±1.36	6.85±1.44	0.049*
Mesial root of second molar	5.80±1.83	5.46±1.95	0.295	4.64±0.92	4.92±0.92	0.074	10.44±1.98	10.38±1.77	0.856
Distal root of second molar	6.95±1.95	6.92±1.85	0.915	3.82±0.62	4.10±0.76	0.020*	10.78±1.94	11.02±1.68	0.42

Means with different superscript letters are statistically significantly different within the same column and parameter*; significant (P≤0.05), SD: standard deviation

The thickness of the lingual bone overlying the roots and the distance between their apices and the mandibular canal is presented in Table 2. The lingual bone was thickest over the distal roots of the first molars (males: 5.58±1.36 mm and females: 4.52±1.24 mm) and thinnest over the distal roots of the second molars (males 3.13±1.50 mm and females: 2.60±1.46 mm). Root apices nearest to the MC were the distal roots of the second molars (males: 1.21±1.45 mm and females: 1.75±1.97 mm), while the furthest were the mesial roots of the first molars (males: 4.00±2.39 mm and females: 4.77±2.58 mm).

**Table 2.** Thickness of the lingual cortex overlaying the mandibular posterior teeth and the distance from their apices to the mandibular canal

Tooth	Lingual cortex to the root			Distance from root apex to the canal		
	(mean±SD, mm)	(mean±SD, mm)
Male	Female	P-value	Male	Female	P-value
First premolar	4.18±1.19	3.98±1.20	0.296	3.07±2.23	3.98±2.01	0.299
Second premolar	4.97±1.40	4.60±1.39	0.097	3.29±2.48	2.88±2.17	0.286
Mesial root of first molar	5.02±1.24	4.28±1.21	<0.001*	4.77±2.58	4.00±2.39	0.067
Distal root of first molar	5.58±1.36	4.52±1.24	<0.001*	4.46±2.60	3.48±2.33	0.021*
Mesial root of second molar	3.26±1.48	3.01±1.37	0.301	2.19±1.98	1.90±2.04	0.383
Distal root of second molar	3.13±1.50	2.60±1.46	0.037*	1.75±1.97	1.21±1.45	0.077

Means with different superscript letters are statistically significantly different within the same column and parameter*; significant (P≤0.05). SD: standard deviation

Statistics concerning MF location are presented in Table 3. It mainly was located between the first and second premolars (51.8%), followed by apical to the second premolar (35.9%), then between the second premolar and the mesial root of the first molar (7.1%). The least common location of the MF was apical to the first premolar (5.3%).

**Table 3.** Summary statistics for demographic data concerning the location of the mental foramen

	Parameter (n=170)	n	%
Mental foramen location	Between first and second premolars	88	51.7
	Between second premolar and first molar	12	7.1
	Inferior to first premolar	9	5.3
	Inferior to second premolar	61	36.0

As regard gender-based differences, the total resection depth (bone thickness+BL root width) was significantly more in males at the first premolar and the distal roots of the first molars (P<0.05). The lingual bone was significantly thinner in the molar area in females (P<0.05). Also, the distance between the root apices and the MC was significantly shorter at the distal root of the first molar in females (P<0.05).

## Discussion

The results of this study provide valuable clinical data essential before surgical intervention. Understanding these measurements will help the operator choose the best surgical approach and prevent unnecessary bone destruction leading to more postoperative complications and delayed or incomplete bony healing (17). This study attempted to measure the bone and root thickness in the BL dimension separately and combined to help clinicians understand the depth needed to locate and resect the root entirely and assess the case difficulty level (16). While locating lingually-positioned roots may be feasible, adequate resection and retro-preparation can be challenging due to the limited accessibility and visibility, as the osteotomy extends posteriorly. In these cases, clinicians may consider other surgical options such as guided surgery, the “bone-lid” technique, or intentional replantation (18-20). The cortical plate's thickness may also help predict the postoperative pain level following endodontic surgery. It has been recently shown that patients with thicker bone covering the apex are significantly more likely to develop severe postoperative pain (21).

The precision and credibility of CBCT in diagnosing spatial relationships between anatomic structures are well documented in the literature (22). Previous studies, however, either lacked a large sample size or used larger fields of view scans, which may affect the visibility of anatomical structures (23). Before CBCT, only cadaver studies could be used to obtain similar information (24). However, cadaver studies do not allow for sufficient sample sizes, normal data distribution, and sufficient numbers of specimens to calculate gender and age differences (24, 25). While some discrepancies may exist between the values calculated using CBCT and direct clinical measurements, they may not be of clinical relevance (26). All measurements of the bone thickness and root dimensions were assessed at the standard resection depth of 3 mm from the root apex, as previously suggested (4, 11, 16). At that level, the preliminary osteotomy is often initiated to access the root. Also, root resection at this level removes most of the lateral and accessory canals (27). Our results showed that the combined BL thickness of the buccal plate and root increases in a posterior direction, supporting the findings of previous studies (4, 11, 20).

In this study, our results showed that the mean buccal bone thickness increased as the tooth became more posteriorly located. The buccal bone supporting the distal root of the second molar was the thickest, with a mean average thickness of 6.9 mm. Various measurements have been reported in the literature regarding the buccal plate thickness opposite to the distal root of the second molars ranging between 6 to 12 mm (4, 11, 22). The difference in results might be due to the methodologies or the populations studied. On the other hand, the buccal bone was remarkably thin over the premolars and the mesial roots of the first molars ranging between 1.2 to 1.5 mm, making them more accessible and predictable for surgical manipulation using microsurgical techniques. At the resection level, the mesial roots of the first molars had the largest BL dimension, while the second premolar was the smallest among all teeth. The mean BL dimension of the mesial roots was also more prominent than the distal roots of all molars. These results agree with previously published data (4, 11, 16).

A shorter distance between the root apex and the MC was noted as the tooth became more posteriorly positioned. The roots of the second molars were the closest to the MC, with 38% of the mesial roots and 54% of the distal roots located ≤1 mm to the MC. Similar findings were reported by former studies (28-30).

In the study herein, the most common horizontal location of the MF was between the first and second premolars (51.8%), followed by being apical to the second premolar (35.9%) with no significant differences in regards to age or sex (P>0.05). These results agree with previously published data for Polish, Nigerian, Kosovarian, and Iranian populations (31-34) and disagree with other studies in Malawian, Zimbabwean, Turkish, Kenyan, and Indian populations, which found that the most typical location of the MF was apical to the second premolars (35-40). These differences can be attributed to the ethnic variances, different sample sizes, and methodologies.

Not all studies investigated gender-based differences. For example, while a significant difference based on gender was found in an Indian population (34), it was reported neither in the Moroccan population (18) nor in this study among the Egyptian population.

The thinnest lingual bone thickness was measured over the distal roots of the mandibular first and second molars. This agrees with Chiona et al. (41) and Aydin et al. (11), even in the numerical range. Although a surgical intervention in the posterior mandible is usually restricted to a buccal approach, limited visibility towards the lingual part of the osteotomy during surgery may result in iatrogenic extension and damage to the lingual plate resulting in a through-and-through lesion and damage to the lingual artery or nerve (42, 43).

Considering gender-related differences, males generally showed a thicker buccal plate of bone compared to females. However, the differences were not statistically significant. Only the distance between the root apices and the MC was significantly shorter at the distal roots of first molars in females (P<0.05) in accordance with Bürklein et al. (44). Therefore, it can also be concluded from this study that CBCT is a reliable tool to determine anatomical measurements needed for surgical intervention. 

The strengths of this study include a large sample size, detailed and reproducible methodology in terms of measurement acquisition and reference planes adjustments, and statistical analysis of gender-based differences, whereas many of the previous studies would often provide only descriptive statistics (10, 12). However, the study has its limitations, as the results only represent the population investigated. Due to the minor variation between patients, it is still more appropriate to consider scanning patients before surgical intervention in the posterior mandible whenever possible or accessible to allow proper assessment and treatment planning.

### Disclosures

**Conflict of interest:** The authors deny any conflict of interest.

**Ethics Committee Approval:** This study was approved by the Ethics Committee of Ain Shams University (Date: 06/08/2020, Number: FDASU-RecEM061705).

**Peer-review:** Externally peer-reviewed.

**Financial Disclosure:** This study did not receive any financial support.

**Authorship contributions:** Concept – S.S., S.A.E.S.; Design – S.S., S.A.E.S.; Supervision – S.S.; Funding - A.T., N.N.N.; Materials - A.T.; Data collection &/or processing – A.T., S.A.E.S.; Analysis and/or interpretation – S.A.E.S., S.S.; Literature search – N.N.N.; Writing – N.N.N.; Critical Review – S.S., A.A.A.
